# High Rates of Tramadol Use among Treatment-Seeking Adolescents in Malmö, Sweden: A Study of Hair Analysis of Nonmedical Prescription Opioid Use

**DOI:** 10.1155/2017/6716929

**Published:** 2017-12-24

**Authors:** Martin O. Olsson, Agneta Öjehagen, Louise Brådvik, Robert Kronstrand, Anders Håkansson

**Affiliations:** ^1^Psychiatry, Department of Clinical Sciences, Lund, Faculty of Medicine, Lund University, 221 00 Lund, Sweden; ^2^Department of Forensic Genetics and Forensic Toxicology, Swedish National Board of Forensic Medicine, Linköping, Sweden; ^3^Division of Drug Research, Linköping University, 581 85 Linköping, Sweden

## Abstract

**Background:**

Nonmedical prescription opioid use (NMPOU) is a growing problem and tramadol has been suggested as an emerging problem in young treatment-seeking individuals. The aim of the present study was to investigate, through hair analysis, NMPOU in this group and, specifically, tramadol use.

**Methods:**

In a study including 73 treatment-seeking adolescents and young adults at an outpatient facility for young substance users, hair specimens could be obtained from 59 subjects. Data were extracted on sociodemographic background variables and psychiatric diagnoses through MINI interviews.

**Results:**

In hair analysis, tramadol was by far the most prevalent opioid detected. Thirty-two percent screened positive for opioids, and of those, all but one were positive for tramadol. Ninety-eight percent reported problematic cannabis use. Significantly more opioid-positive patients also screened positive for other (noncannabis) drugs, compared to nonopioid users. Sixty-four percent fulfilled criteria of DSM-IV psychiatric disorders, other than substance use disorders according to MINI. Fifty-three percent met the symptom criteria count of ADHD above cut-off level.

**Conclusion:**

In the present setting, tramadol, along with high rates of cannabis use, may represent a novel pattern of substance use among young treatment-seeking subjects with problematic substance use and high rates of concurrent psychiatric problems.

## 1. Introduction

Nonmedical prescription opioid use (NMPOU) is defined as the consumption of an opioid medication that is not prescribed to a user or is consumed in a manner not intended by the prescriber (such as tampering, snorting, or injecting). A substantial increase in the use and abuse of prescription drugs has been noted during the past two decades in the US [1] and in the European Union [2, 3]. Zozel and colleagues [4] investigated more than 16,000 identified cases of adolescent (age 13–19 years) prescription drug abuse in the US between 2007 and 2009, where the most frequent opioids were hydrocodone (32%), oxycodone (15%), and tramadol (11%).

Tramadol, classified as a weak opioid, with an analgesic effect similar to that of codeine, has evoked increasing concern for the risk of developing tramadol dependence [5–7] and a risk of serious adverse reactions, including epileptic seizures and fatal intoxications [8–12]. The question of tramadol misuse has been addressed in the Middle East and Africa [13–16], as a problem drug among adolescents and young adults. In many settings, such as the one studied here, cannabis is still the most frequently used illicit drug among adolescents both in population-based surveys [17, 18] and in treatment-seeking samples [19], and tramadol, although primarily a prescription drug, has been described to be associated with more traditional illicit drugs, including cannabis [14]. In Europe, on the other hand, data from the UK have associated tramadol misuse with risk-taking behavior rather than with marginalization and other addictive disorders [20]. In Sweden, Richert and Johnson [21] investigated the illicit use of buprenorphine and methadone among adolescents and young adults and concluded that benzodiazepines and tramadol were used by adolescents to a far greater extent, indicating that tramadol misuse may have increased during the past few years. Data from police authorities in Sweden have shown that tramadol is the second most common seized pharmaceutical drug on the drug scene nationwide in Sweden [22] which lends support to the assumption that the tramadol used in this milieu comes from other sources than prescription.

In addition, Tjäderborn et al. [23] found that tramadol was the third most common pharmaceutical drug among young drug-impaired drivers with mixed substance use, intoxicated with nonprescribed drugs.

Like substance use in general, NMPOU has been described to be associated with psychiatric symptoms [3, 24, 25], and, specifically, it has been demonstrated that a large proportion of tramadol misusers in Egypt [26] had a psychiatric comorbidity.

An objective way of investigating a drug use history is through hair analysis. Drugs circulating in the bloodstream are trapped in the forming hair when it keratinizes and thus produces a temporal map of a person's drug use [27]. Over the years, several applications have been approved for hair analysis in both forensic and clinical work [28–30]. The analysis of opiates and opioids in hair is of particular interest since it may reveal a period of abstinence prior to fatal intoxication suggesting lowered tolerance [31], but it has also been used to investigate compliance in medication showing underreporting of opioid intake in general and especially tramadol [32].

In the present analyses, based on a sample from a treatment study [33], the first aim was to determine the prevalence of NMPOU, including tramadol, and other substances, through drug screening in hair, in treatment-seeking adolescents and young adults. A second aim was to provide a description of prescription opioid users, with special reference to use and misuse of other substances, psychiatric comorbidity, and sociodemographic data. A third aim was to find out whether opioids found in hair analysis were also reported as the problem drug to be addressed in treatment.

## 2. Materials and Methods

### 2.1. Clinical Setting

Maria Malmö is an outpatient treatment facility for adolescents and young adults with substance use disorders and problematic substance use in Malmö, Sweden. The clinic serves the city of Malmö as well as suburban and rural areas surrounding the metropolitan area. Staff from the addiction center, the social services, and child and adolescent psychiatry collaborates in the treatment of patients. Approximately 300–400 patients apply for treatment every year, either through self-referral or through referral from social service and school counsellors, residential treatment centers, and pediatric departments, or directly, through contact from parents and relatives. The facility has an upper age limit of 25 years, without any formal lower age limit, although patients are almost exclusively aged 13 or above.

### 2.2. Study Population

Patients were recruited to the facility from October 1, 2012, to December 31, 2013, taking part in an RCT assessing the potential effects of an Interactive Voice Response with automated personalized feedback on treatment retention and clinical improvement in treatment [33].

**Figure 1:**
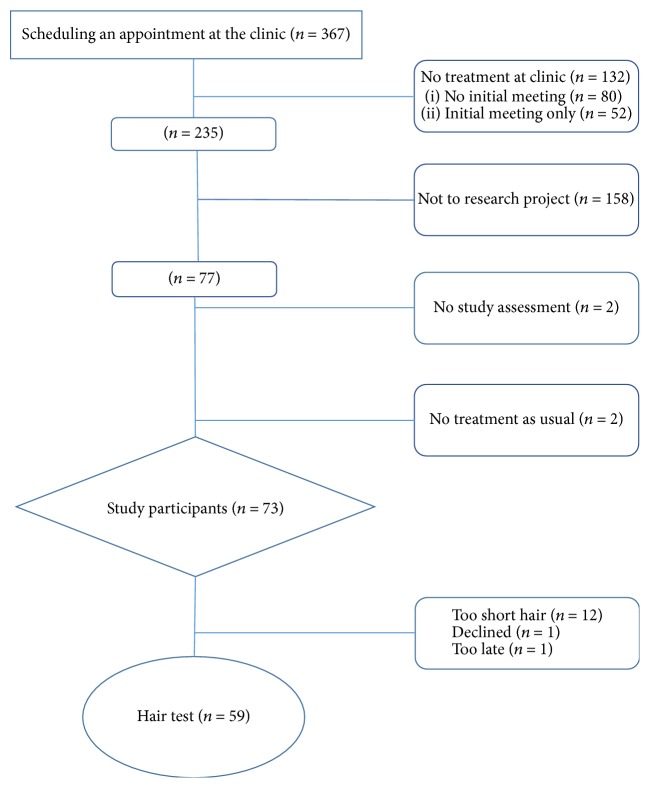
Flow chart of participants in the study.

During the study period from October 2012 to December 2013, 367 patients (30% women) were referred to or applied for treatment at Maria Malmö. The inclusion procedure is presented in Figure 1. Out of 235 patients potentially eligible for the study, 73 patients entered the RCT study, while 158 either were not approached for study recruitment or did not participate. Thus, only 20% of all subjects referred to the facility were included. In order to test the representability of our sample, we performed comparisons between our participants and subjects not included in our study and found no significant differences with respect to gender or the type of facility from which they had been referred. In addition, we also performed a comparison with all subjects applying for treatment the entire following year (2014), which did not demonstrate any significant differences with respect to gender, criminal convictions, and primary drug of abuse.

Out of 73 patients included in the study, 14 patients did not provide a hair specimen. The analysis is therefore based on 59 correctly obtained hair specimens. An attrition analysis showed that, among patients with missing baseline hair analyses, all 14 (100%) were male, and a higher proportion, 10 out of 14 (71%), reported problematic opioid use compared to included patients (21 out of 59, 36%, *p* = 0.019). However, excluded subjects did not differ significantly with respect to age, criminal involvement, MINI psychiatric disorders, or ADHD symptom screen.

### 2.3. Assessment

All patients entering the study were assessed by the staff at the clinic (nurses, social workers) according to standard procedures at the clinic, including the interview instrument Ung-DOK [34], which is used in Sweden at juvenile detention institutions, residential treatment centers, and outpatient facilities for young patients with substance use. The assessment includes questions on sociodemographic factors, self-reported substance use patterns, and self-reported psychiatric symptoms. All patients entering the study had been given information on hair analysis in the patient information and in the form for written consent.

In order to describe the sample, we used data from the Ung-DOK questionnaire for sociodemographic variables, such as sex, age, ethnicity (parents born outside Sweden), criminality (convicted for crime), crime victimization, and reported problems in childhood (psychiatric problems in the patient's family). For data on self-reported “problem drug,” data were extracted from the Ung-DOK questionnaire, defining which drugs were the primary drug and other problem drugs (as defined by both the patient and the therapist). The following drugs are specified as problem drugs in the Ung-DOK questionnaire: (1) alcohol; (2) cannabis; (3) amphetamine; (4) cocaine; (5) ecstasy; (6) LSD; (7) heroin; (8) methadone; (9) buprenorphine; (10) GHB; (11) crack; (12) solvents; (13) benzodiazepines; (14) other sedatives; (15) anabolic androgenic steroids; (16) other drugs (here, patients could specify tramadol or any other drug).

Psychiatric assessment took place through interviews before entering the study, carried out by a research assistant, a mental attendant with a long experience of conducting psychiatric interviews for research in both forensic psychiatry and general psychiatry. Interviews were conducted directly after inclusion in the study before randomization. For psychiatric diagnoses, we used data from the MINI [35] interview. In addition, because of the high prevalence of ADHD among young substance users [36, 37], and as this condition is not assessed in the MINI interview, screening for ADHD symptoms was carried out, using the list of symptom criteria from the DSM-IV [38] interview. Here, however, full clinical assessment, including the assessment of impaired function in different aspects of life and childhood symptom history, could not be carried out.

A hair specimen was obtained by the research assistant according to instructions from the Department of Forensic Toxicology at the National Forensic Center in Linköping, Sweden. Hair was cut close to the scalp accordingly to guidelines [28] and sent to the National Board of Forensic Medicine for analysis. Hair samples were stored in darkness at room temperature prior to analysis. From each hair sample, a 15 mm segment from the root end was used for analysis. This represents a 1- to 1.5-month time window prior to sampling. Extraction of the analytes from the hair matrix was achieved by incubation in methanol : acetonitril : formic acid for 37 hours at 37 degrees C according to a previously validated and published procedure [39]. After incubation, analytes were identified and quantified using high resolution mass spectrometry based on a method modified from a previous published procedure [40]. The method included 10 opioids and several illicit drugs (see Table 1). In addition to traditional illicit drugs, we analyzed common prescription drugs which are more recently known to have abuse potential, such as z drugs [41] and antihistaminergic sedatives [42, 43]. The method also qualitatively included 130 synthetic cannabinoids [44]. The method did not include tetrahydrocannabinol (natural cannabis).

### 2.4. Statistics

SPSS version 21 was used. Descriptive analyses were carried out for the detection of NMPOU, including tramadol, as well as for other substances included in hair analysis. Baseline characteristics for comparison between groups were calculated using Pearson's chi-squared test for categorical variables. Fisher's exact test was used for small group sizes.

### 2.5. Ethical Approval

The study was approved by the Regional Ethics Committee in Lund (file number 2012-217) and has been registered at ClinicalTrials.gov (trial identifier NCT01706380).

## 3. Results

Among included subjects (*n* = 59), 56 percent were male, and the mean age was 18.0 years (std deviation 2.66 years, median 17 years, and interquartile range 16–20).

Out of 59 correctly obtained baseline hair analyses, 19 (32%) specimens were positive for prescription opioids. In all, 18 (31%) patients were positive for tramadol, one of them also positive for methadone. One patient was exclusively positive for codeine. Eleven specimens (19%) were positive for cocaine, five (8%) for synthetic cannabinoids (AM-1220, AM-2201, XLR-11, and 5F-AKB-48), four (7%) for amphetamine, and three (5%) for MDMA. One specimen (2%) was positive for diazepam. The distribution of positive hair specimens for other drugs than opioids is shown in Table 2.

Opioid-positive patients were significantly more likely to be positive for any nonopioid drug, compared to opioid-negative patients: 7/19 (37%) versus 5/40 (13%), *p* = 0.04, Fisher's exact test.

In bivariate analyses, presented in Table 3, opioid-positive and opioid-negative subjects did not differ with respect to crime convictions, age above 18 years, male gender, or psychiatric diagnoses, either for MINI substance-related or non-substance-related psychiatric disorders, or for a positive symptom screen for ADHD.

In the whole sample, 64% (*n* = 38) met diagnostic criteria of at least one MINI psychiatric disorder other than alcohol or substance use disorders (51% affective disorders, 47% anxiety disorders, and 20% psychotic disorders). Eighty and 25 percent fulfilled criteria of drug use disorders and alcohol use disorders, respectively. Also, 53% fulfilled the symptom criteria count for ADHD. Altogether, 80% (*n* = 47) fulfilled either a MINI psychiatric diagnosis (other than an alcohol or drug use disorder) or the ADHD symptom screen. The most common self-reported problem drugs were cannabis (98%), tramadol (34%), cocaine (24%), ecstasy (24%), and synthetic cannabinoids (“spice,” 24%).

Twenty-nine percent reported that one or both parents were born outside Sweden. Thirty-four percent reported having been sentenced for crime; more than half of the sample reported having been victim of crime. Thirty-six percent of the sample reported psychiatric problems in the family during childhood and upbringing, and almost 40% reported alcohol and drug problems in the family.

Opioid detection in hair was significantly correlated with the reporting of opioids as a problem drug. A clear majority of those with opioids detected in hair, 74%, also reported opioids as a problem drug, while 18% in the group without opioids detected (*p* < 0.0001) reported opioids as problem drug. Likewise, tramadol detection was significantly associated with the reporting of tramadol as a problem drug (78% versus 15% in the groups without tramadol detected, *p* < 0.00001).

## 4. Discussion

To the best of our knowledge, this study provides the first example of a clinical application of hair analysis for drug detection in a population of adolescents and young adults.

In the present study on young patients seeking treatment for substance use problems, the main finding was that the predominating opioid misused was, by far, tramadol, in clear contrast to other opioids commonly misused in other settings. Other prescription opioids, such as morphine, oxycodone, or buprenorphine, were not detected, and nor did we detect illicit opioids traditionally seen as “street opioids,” such as heroin (as indicated by an absence of acetylmorphine). Methadone was detected in one patient, also positive for tramadol, whereas only one opioid-positive subject screened positive for codeine. According to recent figures from the same unit, presented in a research application this year, the problem with tramadol is still considerable. During the year 2016, 40% of the adolescents and young adults seeking treatment at the facility had a tramadol problem (personal communication, unit manager Maria Almazidou).

Tramadol as the preferred prescription opioid is in contrast to the reports from the US on the prescription opioid epidemic [1, 4], but might be an expression of a trend towards a more common prescription of tramadol in non-US settings [2]. Tramadol use has been described in Europe among young adults [20] and in the Middle East [13, 14] among adolescents. Our data converge with the findings from Egypt and Iran where researchers have reported results from school surveys indicates a problematic tramadol use, often in combination with cannabis, among students.

Other sources of information are consistent with tramadol representing a novel drug use pattern in the present setting. A police report from Sweden [22] indicates that tramadol and buprenorphine are the most prevalent opioids misused nationwide in Sweden. Still, the finding of tramadol as the predominating and nearly exclusive type of opioid used in the present study, among treatment-seeking adolescents and young adults, is an important finding. Although we have no data on whether the tramadol used was obtained illegally or from prescription, it is highly unlikely that adolescents in this age group should be prescribed tramadol. The clinical impression from staff at the unit (personal communication, unit manager Maria Almazidou) is that the patients report that they obtain the drug illegally, from “street pushers.” Pharmacoepidemiological studies on prescription of opioids among adolescents in the Nordic countries show that tramadol prescription on pain indication is rare in Sweden [45]. Additionally, a national register study on prescription of opioids in Sweden [46] showed that the number of individuals prescribed tramadol has decreased by 54% from 2006 to 2015 in the adult population.

We could also see that problematic use of cannabis, based on self-report data, as demonstrated in nearly all subjects included, was often combined with the use of tramadol. In addition, a majority of those who screened positive in hair analysis for opioids were also positive for other drugs, apart from cannabis, which for technical reasons could not be screened for in the present study. These included synthetic cannabinoids, amphetamine, cocaine, and MDMA, possibly referred to as “club drugs,” rather than heroin and amphetamine, more traditionally seen as “street drugs” often seen as primary drugs of abuse in individuals with severe drug use disorders, including injection drug use [47, 48]. This has been in contrast to the use of cocaine, often considered to be a “street drug” in several other European countries [49], but commonly referred to as a “club drug” in Sweden. In recent years, it has been reported in police data and national survey data that cocaine may be increasingly available on the drug market, along with a sharp decrease in the price of the drug [50–52].

Consistently, Winstock et al. [20] showed, in an Internet survey of more than 7,000 respondents in the UK, that tramadol misuse was more common in a younger and more risk-taking population, differing from more marginalized strata of substance users. Our finding, of almost exclusive misuse of tramadol as a prescription opioid, together with cannabis and other “club drugs,” might be consistent with the observation that tramadol users differ from young individuals using traditional illicit drugs. It also sheds light on the recent anecdotal reporting on social media, as well as media reports [53], indicating a specific drug use pattern combining cannabis and tramadol, something which calls for studies addressing the potentially cooccurring use of these substances in young individuals.

In the present study, treatment-seeking patients were examined at baseline with respect to a large number of psychiatric disorders, including DSM-IV axis I disorders and antisocial personality disorder, and, in addition, patients were screened for ADHD symptoms, although the latter could not be completed with data describing the diagnostic criteria referring to level of impairment and childhood onset. Although psychiatric assessment at baseline should be seen as preliminary and addressed with great caution, preliminary rates of psychiatric comorbidity in this group were high. In this respect, our sample displays the same characteristics as earlier studies on treatment-seeking adolescents [54, 55]. Although the present study is clearly underpowered for more thorough comparisons of comorbidity across different substance classes, subjects screening positive for opioids, nearly exclusively tramadol, did not demonstrate a distinct pattern of comorbidity and at least did not display more extensive comorbidity problems than opioid-negative subjects. For tramadol, it has been reported that this type of drug use may be associated with increased rates of psychiatric comorbidity [26], and tramadol has been suggested as a potentially antidepressant agent [56–59]. Due to the small sample of the present study, this hypothesis could not be tested here, compared to subjects with other substance use patterns. Further research is needed to elucidate the possible comorbidity among tramadol users in larger, population-based samples.

## 5. Limitations and Strengths

An obvious limitation in this study is the high attrition rate and the small size of the sample. Of the original sample of 367 patients referred to the clinic, only 235 persons were eligible. One-hundred and forty-two patients did not turn up to the first appointment or chose to discontinue after the first meeting. Thus, 73 patients entered the RCT study [33]. One possible explanation to the high attrition rate could be the fact that patients had to book separately scheduled meetings with the research assistant before inclusion in the study.

However, we did not observe any significant differences between those who entered the study and those who did not participate. The population also seems to be representative of similar clinical populations [54, 55]. Only 73 patients out of 367 (20% of the whole sample) entered the study, and out of those 59 (81%) took part in the hair analysis. The main reason for the latter was that their hair was too short.

The fact that a majority of the patients, who did not leave any hair specimen, reported tramadol as a problem drug indicates that the prevalence may be even higher, if hair specimens had been available in this group as well. An important limitation is also that cannabis could not be measured in the present hair analysis. We therefore had to rely on self-report from interviews to assess the use of cannabis in the sample.

However, an important strength of the present study is the use of a detailed and exact methodology for drug analysis, which is an important step forward compared to, for example, urine analyses. By using hair analysis, we could get an objective picture of drug use patterns over a period. In addition, the hair analysis could cover drugs that are not screened for in ordinary test panels. Hair analysis detection of tramadol was strongly associated with the self-report of tramadol as a problem substance, such that toxicological analysis in hair clearly could be an important contribution for the detection of tramadol in clinical settings. We propose a more profound investigation on substance use patterns and psychiatric comorbidity among adolescents and young adults using prescription opioids including tramadol.

Not unexpectedly, there were some differences between the hair analyses and self-reports despite good agreement. Tramadol could be detected without being considered a problem drug, if it is casually used for recreational purpose or, although highly unlikely in this population, is prescribed for pain relief.

## 6. Conclusions

In hair analysis of young patients seeking treatment for substance use problems, and for whom problematic cannabis use was reported in nearly all cases, tramadol was the predominating prescription opioid used, indicating a new substance use pattern combining cannabis and “club drugs” with tramadol. Further studies are needed to examine tramadol use in clinical and community populations and to investigate psychiatric disorders in larger groups of tramadol users.

## Figures and Tables

**Table 1:** Substances included in the quantitative method for drugs in hair.

Opioids	Benzodiazepines and benzodiazepine-like medications	Stimulants	Sedatives
Morphine	Diazepam	Amphetamine	Alimemazine
Codeine	7-Amino-clonazepam	Methamphetamine	Hydroxyzine
Acetylmorphine	7-Amino-nitrazepam	Cocaine	Promethazine
Buprenorphine	7-Amino-flunitrazepam	Benzoylecgonine	Propiomazine
Methadone	Alprazolam	MDMA	
Oxycodone	Zopiclone		
Tramadol	Zolpidem		
Ethylmorphine	Zaleplon		
Ketobemidone			
Fentanyl			
Propoxyphene			

**Table 2:** Positive hair specimens in opioid positive and opioid negative patients, *N* = 59 (excluding nonsynthetic cannabinoids).

Positive hair specimens^*∗*^	Opioid positive (*n* = 19)	Opioid negative (*n* = 40)
Tramadol	18	0
Methadone	1	0
Codeine	1	0
Synthetic cannabinoids, “spice”	4	1
Cocaine	3	8
Amphetamine	2	2
MDMA	2	1
Diazepam	0	1
*Patients, positive for any nonopioid drug*	*7*	*5*

^*∗*^A patient could be positive on more than one hair specimen.

**Table 3:** Nonmedical prescription opioid use (NMPOU), detected by hair analysis, with reference to sociodemographic and psychiatric variables (*n* = 59).

	NMPOU positive in hair analysis, *n* (%) *n* = 19	NMPOU negative in hair analysis, *n* (%) *n* = 40	*p* value
*Sociodemographic variables*			
Male sex	11 (58)	22 (55)	0.83
Above 18 years	9 (47)	10 (43)	0.72
Born in Sweden	17 (90)	35 (88)	0.83
Any parent born outside Sweden	8 (42)	9 (23)	0.12
Sentenced for crime	8 (42)	12 (30)	0.36
Victim of crime	11 (58)	21 (53)	0.70
Psychiatric problems in family during upbringing	5 (26)	16 (40)	0.31
*Psychiatric variables*			
MINI, all alcohol use disorders	4 (21)	11 (28)	0.75^1^
MINI, all drug use disorders	16 (84)	31 (78)	0.73
MINI, affective disorders	8 (42)	22 (55)	0.36
MINI, anxiety disorders	6 (32)	22 (55)	0.09
MINI, psychotic disorders	2 (11)	10 (25)	0.30^1^
MINI, antisocial personality disorder	2 (11)	12 (30)	0.19^1^
DSM-IV ADHD symptom count above cut-off	9 (47)	22 (55)	0.58

^1^Fisher's exact test.

## Abbreviations

NMPOU: Nonmedical prescription opioid use
MINI: Mini International Neuropsychiatric Interview
DSM-IV: Diagnostic and Statistical Manual, fourth edition
DOK (DOC): Swedish documentation system.

## Data Access

All data from the study can be accessed from the corresponding author.
